# Effects of First Feed Administration on Small Intestinal Development and Plasma Hormones in Broiler Chicks

**DOI:** 10.3390/ani10091568

**Published:** 2020-09-03

**Authors:** Jiangshui Wang, Dianchun Wang, Kaixuan Li, Lei Xia, Yuanyuan Wang, Lei Jiang, Chianning Heng, Xiuyun Guo, Wei Liu, Xiuan Zhan

**Affiliations:** Key Laboratory of Animal Nutrition and Feed in East China, Ministry of Agriculture and Key Laboratory of Animal Feed and Nutrition of Zhejiang Province, Feed Science Institute, College of Animal Science, Zhejiang University (Zijingang Campus), Hangzhou 310058, China; 11817008@zju.edu.cn (J.W.); 21817058@zju.edu.cn (D.W.); 11517020@zju.edu.cn (K.L.); 21017030@zju.edu.cn (L.X.); 11917027@zju.edu.cn (Y.W.); 21817008@zju.edu.cn (L.J.); 21817419@zju.edu.cn (C.H.); 21717080@zju.edu.cn (X.G.); 21717017@zju.edu.cn (W.L.)

**Keywords:** first feed administration, intestinal development, barrier function, broiler chicks

## Abstract

**Simple Summary:**

In this study, the effects of first feed administration on intestinal morphology, barrier function, and plasma hormones in broilers during the initial 168 h posthatch. Results revealed that early feeding posthatch had a positive effect on small intestinal growth by increasing weight and improving intestinal morphology and barrier function. In other words, early feeding promoted intestinal development, which could be very meaningful for commercial broiler production.

**Abstract:**

(1) Background: Under practical conditions, newly hatched chicks were usually withheld feed and water for 48 to 72 h. It was shown that early feeding after hatch promoted gastrointestinal development of broiler chicks. However, the mechanism of early feeding affecting intestinal development in chicks needs further research. The present study was conducted to investigate the effects of first feed administration on intestinal morphology, barrier function, and plasma hormones in broilers during the initial 168 h posthatch. (2) Methods: A total of 720 one-day-old chicks (newborn chick, Lingnan Yellow) were placed 2 h after hatch and randomly assigned to three treatments: Group A (feed immediately after placement), Group B (fasting for 24 h after placement), and Group C (fasting for 48 h after placement). The trial lasted for 168 h and water ad libitum all the time. Sampling was performed at 0, 24, 48, 72, 120, and 168 h. (3) Results: Higher (*p <* 0.05) absolute weight and relative weight of the small intestine were observed in Group A. Moreover, the villus height, crypt depth, and ratio of the jejunum and ileum were significantly higher (*p <* 0.05) in Groups A and B than those in Group C. Microvilli of the duodenum were closely packed in Group A but sparse and disorganized in Groups B and C. The expression levels of mRNA and protein of tight junction genes (occludin and claudin-1) were upregulated (*p <* 0.05) in Group A. The levels of gastrin and insulin in plasma were decreased (*p <* 0.05) significantly in the Groups B and C. However, chicks in Groups B and C had higher (*p <* 0.05) plasma glucagon levels at 24 and 48 h after placement. (4) Conclusions: These results suggested that early feeding posthatch had a positive effect on small intestinal growth increasing weight and improving intestinal morphology and barrier function.

## 1. Introduction

In the past few decades, the poultry industry has grown rapidly, with poultry consumption increasing globally, and this increase is expected to continue, especially in developing countries [1]. Faster growth and higher feed efficiency have been made during the past decades. Broiler chickens have undergone huge genetic selection pressure for improved growth performance, resulting in drastic changes in feed conversion ratio, which increased nearly 50% over the last few decades [2,3]. A healthy gut is critically important for better growth performance of poultry. Commercial broilers are grown to market weights at ever-decreasing ages so that the first week posthatch represents an increasing proportion of the total growing period, and thus an important period relative to optimizing intestinal growth and development [4]. Newly hatched chickens remain in the incubator until almost all chickens have hatched, after which they are collected [5]. After that, the chickens undergo hatchery treatments, such as vaccination, sex determination, and beak trimming before they are transported to the farm [6].

Under practical conditions, chicks were usually withheld feed and water for 48 to 72 h [5]. Chickens do not have access to feed and water until placement at the farm. The duration of this period depends on the hatch window, hatchery treatments, transportation, and so on. Residual yolk sac forms the only source of energy before the chickens have access to feed. However, although the residual yolk sac is sufficient to maintain the chicks during the first three to four days of postnatal life, it cannot fully support the potential for growth and development of the gastrointestinal and immune system [7]. As the marketing age of broilers decreases, the length of the transition from endogenous yolk dependence to exogenous food utilization becomes increasingly critical [8]. Access to feed and water immediately after hatching is very important for the overall growth performance of poultry [9].

Previous studies have proven the detrimental effects of feed deprivation on broilers with respect to growth performance [10,11,12], immune system [7], gastrointestinal development [13], muscle development [14], and so on. Bigot et al. also suggested that feeding delay posthatch may distort genetic selection by masking the expression of genetic potential and disturbing the estimation of chick breeder value [4]. Undoubtedly, early feeding enhances growth performance [4], improves the nutritional maturity of chicks, increases gastrointestinal development, and has long term metabolic effects [6]. The reason may be that feed intake stimulated the secretion of yolk into the small intestine after hatching and triggered uptake mechanisms of hydrophilic compounds [15]. Actually, the Patio system, in which feed and water are provided immediately after hatching, was developed to overcome the negative effects of early feed deprivation posthatch [16]. This system has been proved to increase organ weights of chicks compared with ordinary hatching systems [17].

The gastrointestinal tract is the largest endocrine organ in the body [18]. Gut hormones function to optimize the process of digestion and absorption of nutrients by the gut [18]. Gastrin and cholecystokinin (CCK) have related physiological roles in vertebrates, being heavily implicated in peripheral signaling to regulate appetite and digestive organ activity, as well as in emotion and behavior [19]. Insulin and glucagon are the most important hormones in the control of glucose homeostasis [20]. However, there is no comprehensive study on the effects of early fasting on plasma hormones. Intestinal epithelial cells serve as a barrier between hostile external environments and the internal milieu [21]. Impaired intestinal barrier function or an increased intestinal permeability (IP) may promote the translocation of bacteria and the entering of allergenic compounds from the gut into the body in pigs after weaning [22]. Growing evidence demonstrated that increases in IP play a pathogenic role in human diseases, such as inflammatory bowel disease and celiac disease, and functional bowel disorders, such as irritable bowel syndrome [23]. Adequate feed intake levels after weaning prevent the loss of the intestinal barrier function [22]. In broilers, previous researches explored the effects of delayed feeding posthatch mainly through assessing IP. However, the intrinsic mechanism of the change of IP is unclear. In the present study, we evaluated the effects of early feed deprivation on intestinal barrier function by testing changes in the intestinal tight junctions using real-time polymerase chain reaction (RT-PCR) and Western blots analysis. In addition, the intestinal morphology was further observed by scanning electron microscopy to explore the mechanism of intestinal development delay in the present study, which could observe the microstructure changes more clearly.

## 2. Materials and Methods 

### 2.1. Animal Ethics

The experimental protocols were in accordance with the Chinese guidelines for animal welfare and approved by the Animal Welfare Committee of the College of Animal Sciences of the University of Zhejiang (No. ZJU2013105002; Hangzhou, China).

### 2.2. Animals and Experimental Design

Hatching eggs were collected from breeders of 31-week-old Lingnan Yellow at a local hatchery (Qunda Breeder Company, Jiaxing, China). The hatching eggs were incubated under regular conditions (37.8 °C, 55% RH). Only 720 chicks hatched within 2 h were collected and randomly allotted to three groups with six replicates of 40 each after weighing. There was no significant difference between each pen of chicks in initial weight and weight distribution. After placement at the farm (Xingjian Culture-Farm, Jiaxing, China), Group A was fed ad libitum immediately and the first feed intake time of Group A was defined as 0 h (corresponding to 4 h posthatch). Groups B and C were delayed access to feed for 24 and 48 h, respectively, after placement, corresponding to 28 and 52 h posthatch. All chicks were reared at floor pens with 5 cm deep fresh wood shavings and were provided with water ad libitum after placement. Diets (Table 1) were formulated to meet the nutrient requirements suggested by NRC 1994 [24]. Chicks were fed ad libitum once they had access to feed. The whole experiment lasted for 8 d with a light schedule of 23 h light and 1 h darkness. The temperature of the chicken house was set at 35 °C on Day 1, and it was then decreased by 0.5 °C each day until Day 8.

### 2.3. Sample Collection

At 0, 24, 48, 72, 120, and 168 h after placement, four chicks from each replicate (24 chicks from each group) were weighted and slaughtered for sampling respectively. Blood samples were obtained from the heart and collected in anticoagulant tubes coated with EDTA. Plasma was then separated by centrifugation of blood samples at 4000 g for 15 min at 4 °C, and stored at −80 °C until subsequent analysis. After blood collection, chicks were slaughtered by cervical dislocation. The small intestine (including duodenum, jejunum, and ileum) of each chick was weighed. About 2 cm segments of the mid regions of the jejunum and ileum were collected, flushed gently with ice-cold PBS (pH 7.4) to remove the intestinal contents and immediately fixed in a 4% formaldehyde solution for histological measurement. An approximately 2 cm segment of the duodenum was fixed with a 2.5% glutaraldehyde solution for analysis of microvillus under a scanning electron microscope. For gene expression and Western blot of tight junction protein, about 5 cm segment of the jejunum was snap-frozen in liquid N_2_ and then stored at −80 °C.

### 2.4. Experimental Parameters Measured

The levels of gastrin, CCK, insulin, and glucagon in plasma were quantified using an enzyme-linked immunosorbent assay kit (Nanjing Jiancheng Bioengineering Institute, Nanjing, China) according to the manufacturer’s protocols.

### 2.5. Intestinal Morphological Analyses

After embedded in paraffin blocks under standard procedures, the jejunum and ileum segments were cut into 5 μm thick sections and then stained with hematoxylin–eosin. Photographs of the stained sections were taken through a Nikon microscope (Nikon Corp., Tokyo, Japan). The measurements of villus height (VH) and crypt depth (CD) were conducted with Image-Pro software (MediaCybernetics, Rockville, MD, USA). VH was measured from the top of the villus to the junction of the villus crypt. The duodenum segment was removed from the 2.5% glutaraldehyde solution after 24 h and washed in phosphate buffer three times and postfixed with 1% osmium tetroxide for 2 h. Then, the tissue was washed three times again in phosphate buffer and dehydrated with a graded series of ethanol (30, 50, 70, 80, 90, 95, and 100%) for 15 min each time. Samples were dried in a critical point using carbon dioxide, finally coated with a 30 nm layer of gold, and observed under a benchtop scanning electron microscope (JMC 500, Nikon, Tokyo, Japan).

### 2.6. Real-Time PCR

Total RNA was extracted from the jejunum using Trizol reagent kit (TaKaRa Biotechnology, Beijing, China), according to the guidelines of the manufacturer. The integrity of isolated RNA was checked by denatured RNA electrophoresis, and the purity and concentration of each RNA sample were determined using a NanoDrop ND-1000 UV spectrophotometer (NanoDrop Technologies, Shanghai, China). One microgram of total RNA was used for cDNA synthesis according to the PrimeScriptTM RT reagent kit (TaKaRa Biotechnology). The reverse transcription condition was set up as follows: 15 min at 37 °C and 5 s at 85 °C. The primer sequences for the occludin and claudin-1 gene synthesized by GENEray Biotechnology (Shanghai, China) are available in Table 2. RT-PCR was carried out using TB Green^®^ Premix Ex TaqTM (Tli RNaseH Plus; TaKaRa Biotechnology) in CFX96 Real-Time PCR Detection System (Bio-Rad). The reaction mixture contained 2 μL complementary DNA, each 0.5 μL of the forward and reverse primers, 12.5 μL TB Green Premix Ex Taq, and 9.5 μL double-distilled water. The PCR procedures were as follows: a prerun at 95 °C for 30 s, forty cycles of denaturation at 95 °C for 5 s, and annealing at 60 °C for 30 s. Melting curve analysis was performed after each RT-PCR assay to confirm the amplification specificity and purity of the PCR product. The 2^−ΔΔCT^ method was used to calculate the relative mRNA expression levels of occludin and claudin-1 compared with 18S ribosomal RNA.

### 2.7. Western Blot Analysis

The protein expressions of occludin and claudin-1 were determined by Western blotting analysis. The total protein of the jejunum tissue was extracted using a total protein extraction kit (Beyotime Biotechnology, Shanghai, China). Twenty micrograms of protein from each group were separated by SDS-PAGE on 10% polyacrylamide gels. Separated proteins were then electrotransferred onto nitrocellulose membranes (Beyotime Biotechnology). Membranes were blocked with 5% nonfat dry milk in the TBS-Tween 20 buffer. Next, the membrane was incubated overnight at 4 °C with primary antibody. The primary antibodies were mouse monoclonal anti-claudin-1 (Huaan Biotechnology, Hangzhou, China; 1:500) and rabbit polyclonal anti-occludin (Huaan Biotechnology; 1:1000). Membranes were subsequently probed with rabbit monoclonal anti-beta actin (Abcam, Cambridge, UK; 1:1000). The secondary antibodies were goat anti-Mouse IgG Antibody (Thermo Fisher Scientific, Waltham, MA, USA) and goat anti-rabbit IgG antibody (Thermo Fisher Scientific, Massachusetts, USA). Membranes were incubated with an enhanced chemiluminescence detection kit (Beyotime Biotechnology). Images were taken (GS-700, Bio-Rad, Hercules, CA, USA) and were quantified by densitometry with Image J software.

### 2.8. Statistical Analysis

Data from the present study were analyzed by one-way ANOVA using SPSS statistical software (version 20.0 for windows; SPSS Inc., Chicago, IL, USA). The replicate of four birds for plasma hormone levels and intestinal weight, but the individual chick for RT-PCR and Western blot was set as the experimental unit. Differences among treatments were examined using Tukey’s multiple range tests, and a probability of *p* < 0.05 was considered to be significant. Data were presented as means with the standard errors of the means. Figures were made by GraphPad Prism 7.00 software.

## 3. Results

### 3.1. Small Intestinal Weight

**Figure 1:**
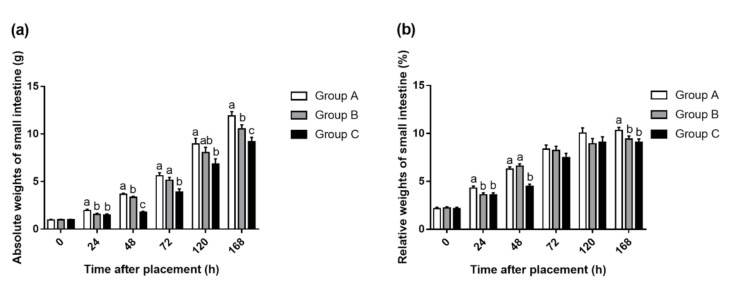
Absolute weights (**a**) and relative weights (**b**) of the small intestine in Group A (fed immediately), B (first feed administered for 24 h), and C (first feed administered for 48 h). Mean values with their SEM (n = 6). (a–c) Mean values within a row with unlike letters were significantly different (*p* < 0.05).

The results of absolute weight of the small intestines suggested that Group A had the highest weight than the other two groups, followed by Group B, and finally Group C (Figure 1a, *p* < 0.05), thereinto no difference was observed between Groups A and B at 72 and 120 h. Additionally, the highest relative weight of the small intestine was still observed at Group A (Figure 1b, *p* < 0.05). During the fasting period, the relative weight of the small intestine in Groups B and C was significantly lower than that of Group A (*p* < 0.05), and then increasing to the same level of Group A at 48 and 72 h, respectively, after refeeding. It was not until 168 h that Group A was significantly higher than Groups B and C in the relative weight of the small intestine (*p* < 0.05).

### 3.2. Intestinal Morphology

**Figure 2:**
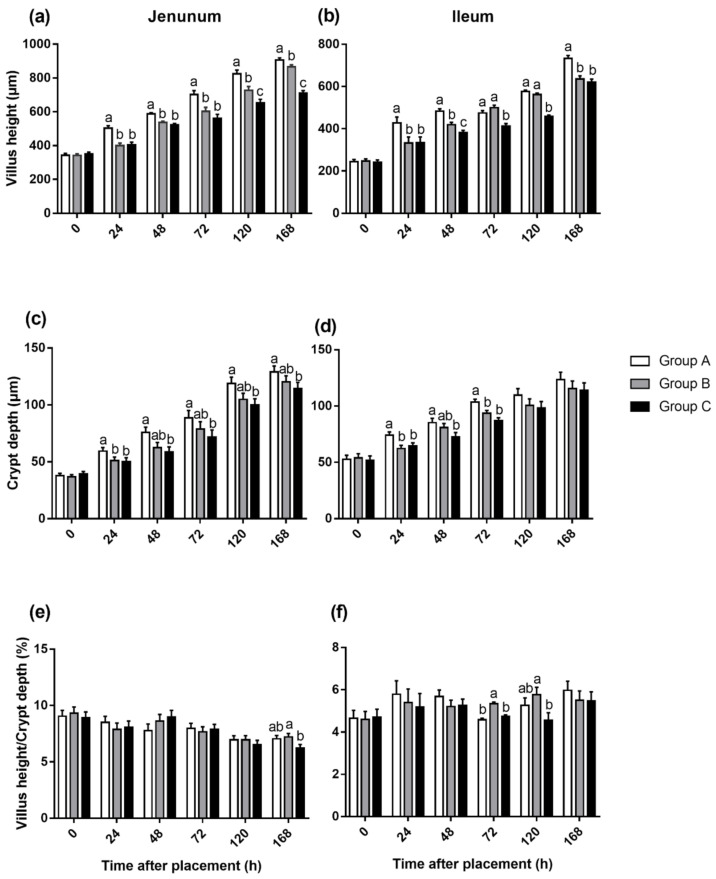
Jejunum and ileum villus height (**a**,**b**), crypt depth (**c**,**d**) and villus height: crypt depth ratio (**e**,**f**) of Group A (fed immediately), B (first feed administered for 24 h), and C (first feed administered for 48 h). Mean values with their SEM (n = 6). (a–c) Mean values within a row with unlike letters were significantly different (*p* < 0.05).

**Figure 3:**
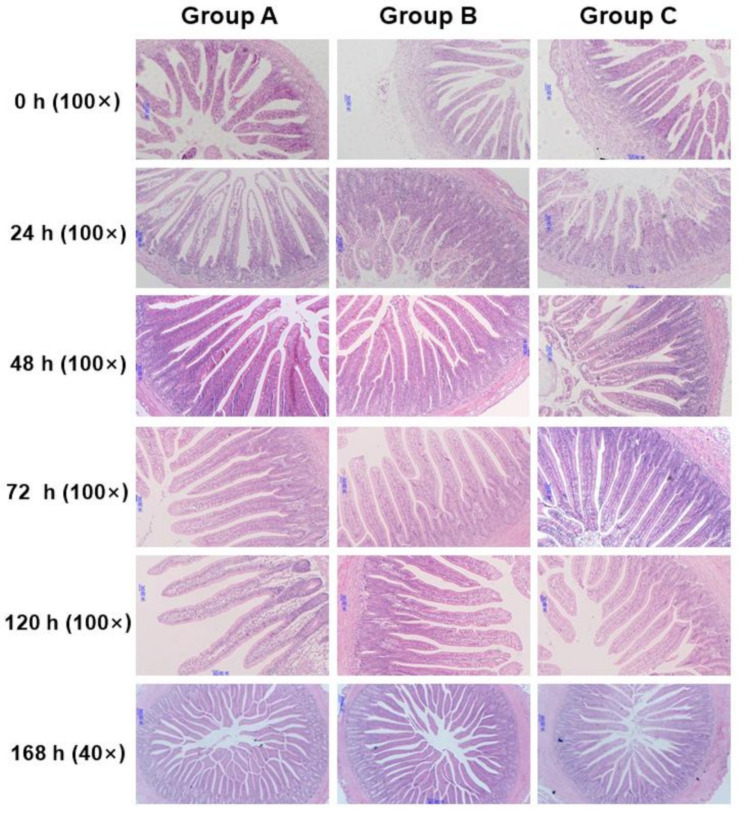
Light microscopy of the cross-sections of the jejunum on Group A (fed immediately), B (first feed administered for 24 h) and C (first feed administered for 48 h) at 0, 24, 48, 72, 120, and 168 h after placement (168 h: ×40, scale bar = 80 mm; others: ×100, scale bar = 30 mm).

**Figure 4:**
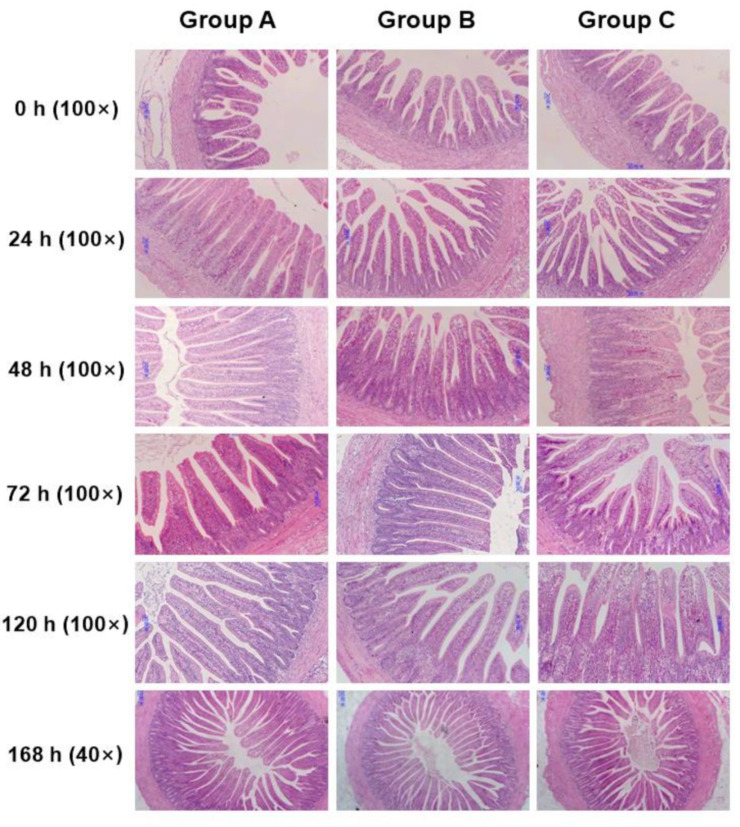
Light microscopy of the cross-sections of the ileum on Group A (fed immediately), B (first feed administered for 24 h) and C (first feed administered for 48 h) at 0, 24, 48, 72, 120, and 168 h after placement (168 h: ×40, scale bar = 80 mm; others: ×100, scale bar = 30 mm).

**Figure 5:**
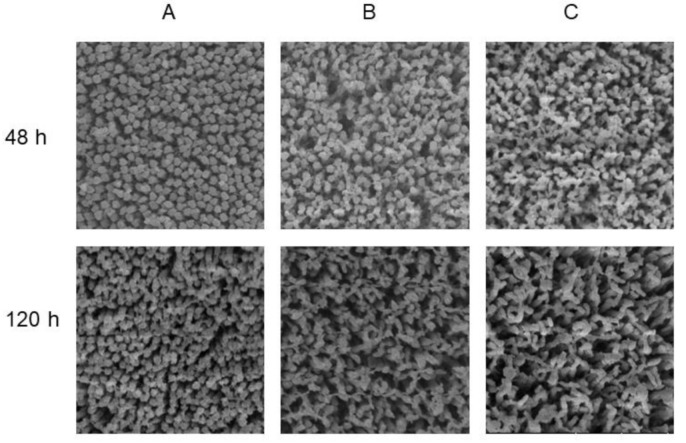
Scanning electron-photomicrograph of the duodenum mucosa microvillus on Group A (fed immediately), B (first feed administered for 24 h) and C (first feed administered for 48 h) at 48 and 120 h after placement (×40,000).

The VH of the jejunum in Groups B and C was significantly lower (*p* < 0.05) than that in Group A from 24 to 168 h after placement, moreover, Group C had lower (*p* < 0.05) VH than Group B at 120 h (Figure 2a). In the ileum, the VH of Group A was also increased by early feeding (Figure 2b). The result in the ileum was similar to the jejunum, which VH of chicks fed immediately were significantly higher (*p* < 0.05) than that of chicks delaying feed, except that there was no difference between Groups A and B at 72 and 120 h. When it came to CD, Group C always had the lowest (*p* < 0.05) CD in the jejunum from 24 to 168 h, but there was no significant difference between Groups A and B except at 24 h (Figure 2c). In the ileum, the difference was only observed at 24, 48, and 72 h. Group A had the highest CD among the three groups (*p* < 0.05), but there was no significant difference between Groups B and C (Figure 2d). In addition, it was observed that Groups A and B had higher villus height: crypt depth ratio (VCR) of the jejunum than that of Group C on 168 h after placement (Figure 2e). In the ileum, however, Group B had the best performance on VCR compared with the other two groups on 72 h (Figure 2f). Figure 3 and Figure 4 are light microscopies of the cross-sections of the jejunum (Figure 3) and ileum (Figure 4) on Groups A, B and C at 0, 24, 48, 72, 120, and 168 h after placement. On 120 h, Groups A and B had higher (*p* < 0.05) VCR compared with Group C. Scanning electron-photomicrograph of the duodenum mucosa of chicks at 48 and 120 h showed that microvilli were closely packed in Group A, and sparse and disorganized microvilli were observed in Groups B and C (Figure 5).

### 3.3. mRNA Level and Protein Expression of Intestinal Tight Junction

**Figure 6:**
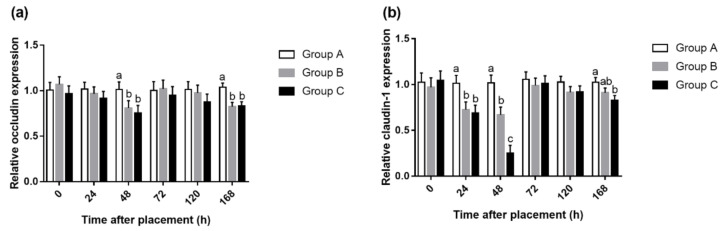
Relative gene expressions of occludin (**a**) and claudin-1 (**b**) in the jejunum on three groups at 0, 24, 48, 72, 120, and 168 h after placement. Values are means and SEM represented by vertical bars (n = 6). (a–c) Mean values with unlike letters were significantly different (*p* < 0.05).

**Figure 7:**
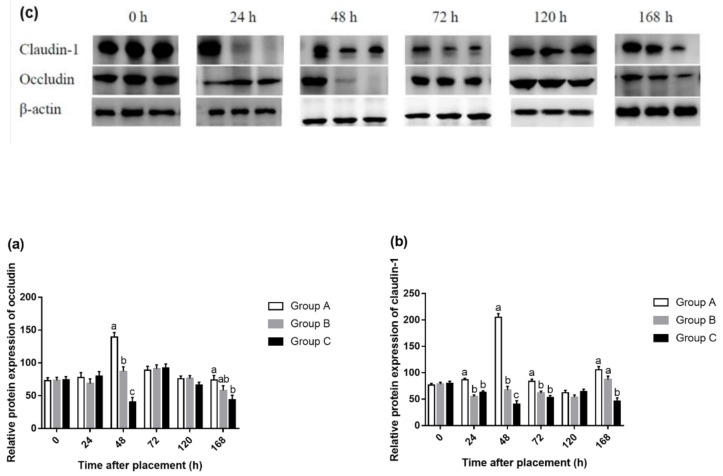
Relative gene expressions of occludin (**a**) and claudin-1 (**b**) in the jejunum on three groups at 0, 24, 48, 72, 120, and 168 h after placement. (**c**) are representative blots of occludin, claudin-1, and β-actin at 0, 24, 48, 72, 120, and 168 h after placement. Values are means and SEM represented by vertical bars (n = 6). (a–c) Mean values with unlike letters were significantly different (*p* < 0.05).

The difference of occludin mRNA level in the jejunum mucosa was only observed at 48 and 168 h after placement (Figure 6). Compared with Group A, feed deprivation for 48 h decreased (*p* < 0.05) mRNA abundances of occludin, but there was no significant difference between Groups A and B. Different from occludin gene, feeding delayed for 24 or 48 h, both reduced gene expression of claudin-1 at 24, 48 and 168 h, when compared to Group A. Moreover, Group C had lower (*p* < 0.05) claudin-1 mRNA level than Group B at 48 h after placement. Fasting for 24 and 48 h decreased (*p* < 0.05) protein expression of occludin at 48, 168 h and claudin-1 at 24, 48, 72 and 168 h, compared with Group A in the jejunum (Figure 7). However, there was no significant difference in occludin and claudin-1 protein levels between Groups B and C.

### 3.4. Plasma Hormone Levels

As shown in Table 3, feeding delay for 48 h posthatch downregulated plasma gastrin level (*p* < 0.05) compared with chicks in Groups A and B, while no difference was observed between Groups A and B at 48 h. After refeeding, the gastrin level in both B and C group was increased to the same level of Group A at 48 h and 72 h, respectively, and then decreased to a lower level than Group A (*p* < 0.05) at 72 h and 120 h, which finally, returning to normal level at 120 h and 168 h, respectively. However, Group A always had the highest level of plasma gastrin (*p* < 0.05). Different from gastrin, the concentration of CCK was not affected by feed deprivation of 24 or 48 h during the whole experiment (Table 4). The difference in plasma glucagon level was only observed at 24 and 48 h (Table 5). Chicks that delayed feed for 24 and 48 h had higher plasma glucagon levels compared with feeding chicks immediately at 24 and 48 h (*p* < 0.05), and then decreased to the same level with Group A for 72 h after placement. After 24 h feed consumption in Groups B and C, the concentration of plasma insulin decreased significantly than Group A at 48 and 72 h, respectively (Table 6). Moreover, the decrease of insulin level sustained until 72 h in Group B, while Group C still had lower plasma insulin levels than the other two groups until 168 h (*p* < 0.05).

## 4. Discussion

In mammals, gastrin and CCK are known as gut hormones to suppress appetite [25]. The central administration of gastrin strongly inhibited food intake and clearly delayed food passage in neonatal chicks [26]. In our experiment, all hungry chicks, before feed access in Groups B and C, had lower plasma gastrin levels due to higher appetite. After refeeding, the appetite was decreased so as to increase the concentration of gastrin in plasma. CCK in the duodenum plays an important role in coordinating food digestion and nutrient absorption by controlling the motility of the gastrointestinal tract and enzyme secretion [27]. Furuse et al. suggested that the suppressive effect of food intake of gastrin/CCK family may be dependent upon the length of the amino acid sequence, as evidenced by the fact that the effect of CCK-8S was weak, but CCK-33S strongly inhibited food intake of chicks [28]. Plasma CCK levels decreased rapidly due to food deprivation for up to five days in male rats, and after only 1 day of refeeding, levels of plasma CCK was restored to control levels [29]. There was no difference in plasma CCK concentration after 24 or 48 h of fasting after placement. That means feeding delay had no effect on plasma CCK concentration in this experiment. The residual yolk sac during the fasting period may contribute to a stable CCK level. Additionally, 48 h may not be long enough to affect plasma CCK concentration. We need further research to explore the mechanism. Shiraishi et al. found that plasma insulin concentration of the male layer and broiler chicks significantly decreased after 24 h of fasting [30]. In our experiment, we also found similar results that early feeding increased plasma insulin concentration from 48 to 168 h in broiler chicks. The reason may be that blood glucose increased after refeeding, and then insulin was secreted to promote glycogen synthesis so as to decrease blood glucose. Conversely, glucagon concentration was enhanced during the fasting period and fell back to a stable level after refeeding in this experiment. This discrepancy between insulin and glucagon in plasma identified with previous studies in which developmental changes in plasma insulin contrasted with glucagon until Day 9 after hatch [31].

In the posthatch period, the small intestine continues to increase in weight more rapidly than the rest of body mass [32]. The small intestine development was ongoing regardless of fasting or not after hatching. Direct feed access resulted in a higher relative weight of the jejunum at Day 4 compared with delayed feed access [33]. Chicks that were subjected to 18 and 36 h of fasting after placement had lower biometrical values for the small intestine [13]. Early feeding increased the weight of intestinal fragments from one to four days of age [4]. Feed supply promoted greater duodenum and ileum weights compared with those of broiler chicks that did not receive feed during the initial 24 h after hatching [34]. Our results were consistent with previous studies except at 72 and 120 h that the relative weight of the small intestine (both males and females) in delayed feeding chicks was restored to the same level of Group A. We believe this phenomenon can be attributed to compensatory growth in delayed-fed chicks. In the present study, we also found that the residual yolk sac was used faster in immediately fed chicks than in feeding delayed chicks (Figure S1). This may be responsible for a higher intestinal weight of chicks fed immediately after placement. The possible reason is that the intestinal motility of fed chicks promoted the transfer of yolk [35]. In addition, early feeding (Group A) increased BW of hatchlings until 168 h posthatch in our experiment (Figure S2), which was in accordance with residual yolk sac results. It is indicated that early feeding promoted yolk sac absorption, intestinal development, and body growth of broiler chicks until 168 h posthatch.

Intestinal morphology has become a common research tool for assessing nutritional effects on the intestine [36]. The small intestine of newly hatched chicks is immature and undergoes morphological, biochemical, and molecular changes during the two weeks posthatch, and the most dramatic changes occur during the first 24 h posthatch [32]. The absence of feed during this time led to a depression in intestinal development. Poults of delayed access to feed depressed the growth rate of villi and enterocyte length in all intestinal segments until 6 d posthatch [37]. Mahmoud and Edens found that feed delayed to newly hatched chicks from the young breeder flock shortened villi, decreased CD and villus surface area in the duodenum through the first week posthatch [38]. Chicks with access to feed had higher VH and CD compared with the fasting group [13,26]. In the current study, the biometric results in the jejunum and ileum are completely compatible with these reports, with a significant effect of fasting on decreasing VH and CD. We also observed a decrease of VCR on 168 h in the jejunum and 120 h in the ileum in Group C. This is consistent with a previous study, in which chicks fed immediately had higher VCR than that of delayed feeding chicks [38]. The absence of nutrients provided by exogenous feed may be responsible for changes in the morphology of the intestinal mucosa. Besides, we also found that feeding delay caused sparse and disorganized microvilli in duodenum mucosa through scanning electron-photomicrograph at 48 and 120 h after placement. This phenomenon is consistent with the morphology results of the jejunum and ileum. Previous studies also identified with the current study. Uni et al. stated that morphological changes following delayed access to feed included some clumping of microvilli and abnormal crypt structure [39]. Chicks’ lack of feed or water showed a higher number of microvillus per area due to a reduction in size when compared with feed and water ad libitum treatments [26]. Therefore, these observations confirmed that the residual yolk sac was not enough to fully support the development of the small intestines.

Fewer studies show the changes in barrier function following delayed access to feed posthatch in broilers. Horn et al. found that 24 h water deprivation reduced occludin gene expression of the jejunum 1 d postweaning and claudin-1 and ZO-1 gene expression of the ileum 7 d postweaning [40]. They also demonstrated that the expression of the tight junction genes ZO-1 and OC were reduced due to food and water deprivation 1 d after weaning [41]. Our study found that expressions of genes occludin and claudin-1 in the jejunum decreased due to 24 or 48 h fasting after placement. This suggested that the yolk sac was not sufficient to support the development of an intestinal tight junction during the initial periods posthatch in broilers. The gastrointestinal epithelium forms the boundary between the body and external environment. It effectively provides a selectively permeable barrier that limits the permeation of luminal noxious molecules, such as pathogens, toxins, and antigens, while allowing the appropriate absorption of nutrients and water [42]. Tight junctions form continuous intercellular contacts controlling solute movement through the paracellular pathway across epithelia [43]. Gilani et al. concluded that fasting periods of 4.5 and 9 h increased IP compared to nonfasted chicks [44]. Fasting significantly increased IP in 21-day-old broiler chickens [45]. In this regard, our results are not contradictory to previous studies. In the present study, feed deprivation for 24 or 48 h decreased protein expression of occludin at 48 and 168 h and claudin-1 at 24, 48, 72, and 168 h in the jejunum. This may suggest that the absence of feed during the initial period posthatch can increase IP. The results of gene expression were basically consistent with that of protein expression, except that there was no difference in gene expression at 72 h, but the protein expression of claudin-1 was significantly higher in Group A compared with Groups B and C. This suggested that early fasting had a more sustained effect on protein expression than gene expression, although mRNA expression is not always necessarily consistent with protein expression.

## 5. Conclusions

In conclusion, our results suggest that early feeding could regulate appetite by affecting the gastrointestinal hormone and pancreatic hormone. Moreover, early feeding could promote intestinal development by increasing intestinal weight, improving morphometric traits of the intestinal mucosa, upregulating the mRNA, and protein expression of tight junctions.

## Figures and Tables

**Table 1:** Composition and nutrient levels of the basal diets for broilers (air-dry basis).

Items	1–8 d
Ingredients (%)	
Corn	54.70
Wheat	5.00
Soybean meal	29.00
CGM	6.00
Soybean oil	1.00
NaCl	0.30
CaHPO4	1.70
Limestone	1.30
Premix *	1.00
Total	100.00
Nutrient levels † (%)	
ME (kJ/kg)	12,171
CP	20.96
Lys	1.10
Met	0.50
Met+Cys	0.85
Ca	0.99
TP	0.66

CGM, corn gluten meal; ME, metabolizable energy; CP, crude protein; TP, total phosphorus. * Supplied per kg of diet: vitamin A, 9600 IU; vitamin D3, 2700 IU; vitamin E, 36 mg; vitamin K3, 3.0 mg; vitamin B1, 3.0 mg; vitamin B2, 10.5 mg; vitamin B6, 4.2 mg; vitamin B12, 0.03 mg; folic acid, 1.5 mg; nicotinamide, 60 mg; D-calcium pantothenate, 18 mg; biotin, 0.225 mg; choline chloride, 1000 mg; Fe, 80 mg; Cu, 8.0 mg; Mn, 80 mg; Zn, 60 mg; I, 0.35 mg; Se, 0.15 mg. † ME is a calculated value, other nutrient levels are measured values.

**Table 2:** Primers used for the genes.

Genes	Forward	Reverse
18S ribosomal RNA	5′-ATTCCGATAACGAACGAGACT-3′	5′-GGACATCTAAGGGCATCACA-3′
Occludin	5′-TCATCGCCTCCATCGTCTAC-3′	5′-TCTTACTGCGCGTCTTCTGG-3′
Claudin-1	5′-TGGAGGATGACCAGGTGAAGA-3′	5′-CGAGCCACTCTGTTGCCATA-3′

**Table 3:** Effects of feed deprivation on plasma gastrin levels in broiler chicks (ng/L).

Time after Placement (h)	Time of Feed Deprivation (h)			SEM	*p*-Value
	0	24	48		
0	76.63	74.08	73.04	6.941	0.869
24	147.4	161.0	159.2	9.191	0.306
48	274.4 ^a^	254.2 ^a^	157.3 ^b^	11.24	0.000
72	245.0 ^a^	146.3 ^b^	256.9 ^a^	20.80	0.000
120	222.7 ^a^	204.4 ^a^	144.2 ^b^	11.37	0.000
168	191.1	209.4	210.0	8.639	0.075

Mean values with their SEM (n = 6). ^a,b^ Mean values within a row with unlike letters were significantly different (*p* < 0.05).

**Table 4:** Effects of feed deprivation on plasma cholecystokinin (CCK) levels in broiler chicks (ng/L).

Time after Placement (h)	Time of Feed Deprivation (h)			SEM	*p*-Value
	0	24	48		
0	110.1	118.2	115.6	5.463	0.350
24	181.6	166.7	168.7	12.81	0.469
48	195.3	187.0	198.9	5.446	0.115
72	203.8	206.8	192.3	10.32	0.362
120	181.0	183.5	194.6	10.75	0.426
168	199.7	186.6	185.5	9.153	0.256

Mean values with their SEM (n = 6).

**Table 5:** Effects of feed deprivation on plasma glucagon levels in broiler chicks (ng/L).

Time after Placement (h)	Time of Feed Deprivation (h)			SEM	*p*-Value
	0	24	48		
0	184.6	189.5	190.8	15.32	0.912
24	156.4 ^b^	216.3 ^a^	223.3 ^a^	10.24	0.000
48	182.4 ^b^	272.1 ^a^	296.3 ^a^	23.34	0.000
72	209.7	205.1	210.4	10.04	0.851
120	212.7	206.8	214.5	8.685	0.657
168	199.9	196.7	207.7	9.375	0.501

Mean values with their SEM (n = 6). ^a,b^ Mean values within a row with unlike letters were significantly different (*p* < 0.05).

**Table 6:** Effects of feed deprivation on plasma insulin levels in broiler chicks (mIU/L).

Time after Placement (h)	Time of Feed Deprivation (h)			SEM	*p*-Value
	0	24	48		
0	33.56	35.47	33.76	3.261	0.816
24	32.91	31.53	32.08	2.358	0.842
48	40.84 ^a^	28.46 ^b^	37.17 ^a^	3.124	0.004
72	37.90 ^a^	24.85 ^b^	28.40 ^b^	2.556	0.000
120	32.95 ^a^	29.09 ^a^	19.72 ^b^	2.147	0.000
168	34.73 ^a^	29.25 ^a^	17.78 ^b^	2.630	0.000

Mean values with their SEM (n = 6). ^a,b^ Mean values within a row with unlike letters were significantly different (*p* < 0.05).

## Supplementary Materials

The following are available online at https://www.mdpi.com/2076-2615/10/9/1568/s1, Table S1. Effects of feed deprivation on absolute weights of the small intestine in broiler chicks (g); Table S2. Effects of feed deprivation on relative weights to the small intestine in broiler chicks (%); Table S3. Effects of feed deprivation on villus height of jejunum in broiler chicks (μm); Table S4. Effects of feed deprivation on villus height of ileum in broiler chicks (μm); Table S5. Effects of feed deprivation on crypt depth of jejunum in broiler chicks (μm); Table S6. Effects of feed deprivation on crypt depth of ileum in broiler chicks (μm); Table S7. Effects of feed deprivation on VCR of jejunum in broiler chicks; Table S8. Effects of feed deprivation on VCR of ileum in broiler chicks; Table S9. Relative gene expressions of occludin in the jejunum on three groups at 0, 24, 48, 72, 120, and 168 h after placement; Table S10. Relative gene expressions of occludin (a) and claudin-1 (b) in the jejunum on three groups at 0, 24, 48, 72, 120, and 168 h after placement; Table S11. Relative protein expressions of occludin in the jejunum on three groups at 0, 24, 48, 72, 120, and 168 h after placement; Table S12. Relative protein expressions of claudin-1 in the jejunum on three groups at 0, 24, 48, 72, 120, and 168 h after placement; Table S13. Effects of initial feeding time on growth performance and feed utilization in broilers; Figure S1. Effects of feed deprivation on relative weight of residual yolk in broiler chicks (n = 6); Figure S2. Effects of feed deprivation on body weight. Mean values with their SEM (*n* = 6).
