# Supplementary material for: Effects of First Feed Administration on Small Intestinal Development and Plasma Hormones in Broiler Chicks
Source: Animals (Basel). 2020 Sep 3;10(9):1568. doi: 10.3390/ani10091568 (PMC7552185; doi:10.3390/ani10091568)
Supplement: Supplementary file 1 [file animals-10-01568-s001.pdf]

Table S1. Effects of feed deprivation on absolute weights of the small intestine in broiler chicks (g)

Time after placement (h)	Time of feed deprivation（h）			SEM
	0	24	48	
0	0.974	0.981	0.975	0.052
24	1.97[a]	1.55[b]	1.48[b]	0.119
48	3.67[a]	3.34[b]	1.78[c]	0.106
72	5.61[a]	5.14[a]	3.92[b]	0.309
120	8.97[a]	8.05[ab]	6.84[b]	0.556
168	11.91[a]	10.53[b]	9.21[c]	0.432

Mean values with their SEM (n 6). a,b,c Mean values within a row with unlike letters were significantly different (P < 0·05).

Table S2. Effects of feed deprivation on relative weights to the small intestine in broiler chicks (%)

Time after placement (h)	Time of feed deprivation（h）			SEM
	0	24	48	
0	2.16	2.22	2.16	0.124
24	4.29[a]	3.60[b]	3.59[b]	0.211
48	6.28[a]	6.60[a]	4.47[b]	0.230
72	8.36	8.22	7.48	0.437
120	10.04	8.93	9.10	0.541
168	10.30[a]	9.40[b]	9.09[b]	0.330

Mean values with their SEM (n= 6). a,b Mean values within a row with unlike letters were significantly different (P < 0·05).

Table S3. Effects of feed deprivation on villus height of jejunum in broiler chicks (μm)

Time after placement (h)	Time of feed deprivation（h）			SEM
	0	24	48	
0	342.9	341.9	350.8	10.44
24	502.8[a]	400.6[b]	405.1[b]	15.20
48	587.5[a]	537.3[b]	523.3[b]	7.444
72	702.1[a]	602.4[b]	562.0[b]	23.66
120	824.8[a]	727.3[b]	652.0[c]	21.95
168	906.1[a]	865.3[b]	712.0[c]	13.00

Mean values with their SEM (n 3). a,b,c Mean values within a row with unlike letters were significantly different (P < 0·05).

Table S4. Effects of feed deprivation on villus height of ileum in broiler chicks (μm)

Time after placement (h)	Time of feed deprivation（h）			SEM
	0	24	48	
0	244.1	247.8	242.0	10.00
24	427.7[a]	333.0[b]	334.0[b]	27.77
48	482.9[a]	419.1[b]	381.0[c]	11.28
72	474.2[a]	500.1[a]	412.6[b]	11.77
120	576.5[a]	562.5[a]	458.2[b]	6.319
168	732.9[a]	636.0[b]	620.9[b]	14.03

Mean values with their SEM (n 3). a,b,c Mean values within a row with unlike letters were significantly different (P

< 0·05).

Table S5. Effects of feed deprivation on crypt depth of jejunum in broiler chicks (μm)

Time after placement (h)	Time of feed deprivation（h）			SEM
	0	24	48	
0	37.97	36.76	39.57	1.959
24	59.30^{a}	51.10^{b}	50.37^{b}	3.224
48	75.93^{a}	62.47^{ab}	58.70^{b}	4.535
72	88.60^{a}	78.80^{ab}	71.57^{b}	6.450
120	119.0^{a}	104.8^{ab}	100.0^{b}	5.437
168	128.9^{a}	120.2^{ab}	114.4^{b}	5.342

Mean values with their SEM (n 3). a,b Mean values within a row with unlike letters were significantly different (P < 0·05).

Table S6. Effects of feed deprivation on crypt depth of ileum in broiler chicks (μm)

Time after placement (h)	Time of feed deprivation（h）			SEM
	0	24	48	
0	52.67	53.93	51.97	3.699
24	74.21^{a}	62.37^{b}	64.60^{b}	2.730
48	85.33^{a}	80.77^{ab}	72.70^{b}	3.690
72	103.6^{a}	93.74^{b}	87.02^{b}	2.490
120	109.7	100.6	98.20	5.729
168	123.4	115.6	113.9	6.606

Mean values with their SEM (n 3). a,b Mean values within a row with unlike letters were significantly different (P < 0·05).

Table S7. Effects of feed deprivation on VCR of jejunum in broiler chicks

Time after placement (h)	Time of feed deprivation（h）			SEM
	0	24	48	
0	9.04	9.33	8.90	0.534
24	8.49	7.89	8.07	0.548
48	7.77	8.62	8.98	0.583
72	7.97	7.66	7.88	0.451
120	6.95	6.96	6.53	0.370
168	7.04^{ab}	7.21^{a}	6.24^{b}	0.308

VCR=villus height: crypt depth ratio

Mean values with their SEM (n 3). a,b,c Mean values within a row with unlike letters were significantly different (P < 0·05)

Table S8. Effects of feed deprivation on VCR of ileum in broiler chicks

Time after placement (h)	Time of feed deprivation（h）			SEM
	0	24	48	
0	4.65	4.60	4.70	0.380
24	5.78	5.39	5.18	0.644
48	5.68	5.20	5.25	0.309
72	4.58^{b}	5.34^{a}	4.74^{b}	0.079
120	5.27^{ab}	5.77^{a}	4.56^{b}	0.349

168	5.97	5.51	5.47	0.436

VCR=villus height: crypt depth ratio

Mean values with their SEM (n 3). a,b,c Mean values within a row with unlike letters were significantly different (P < 0·05)

Table S9. Relative gene expressions of occludin in jejunum on three groups at 0, 24, 48, 72, 120, 168 h after placement.

Time after placement (h)	Time of feed deprivation（h）			SEM
	0	24	48	
0	1.007	1.068	0.968	0.085
24	1.012	1.000	0.954	0.097
48	1.013^{a}	0.864^{b}	0.760^{b}	0.066
72	1.005	1.020	0.949	0.097
120	1.016	0.976	0.878	0.085
168	1.038^{a}	0.826^{b}	0.833^{b}	0.046

Mean values with their SEM (n 6). a, b, c Mean values within a row with unlike letters were significantly different (P < 0·05).

Table S10. Relative gene expressions of occludin (a) and claudin-1 (b) in jejunum on three groups at 0, 24, 48, 72, 120, 168 h after placement.

Time after placement (h)	Time of feed deprivation（h）			SEM
	0	24	48	
0	1.024	0.971	1.047	0.103
24	1.016^{a}	0.728^{b}	0.692^{b}	0.084
48	1.018^{a}	0.669^{b}	0.252^{c}	0.085
72	1.058	0.991	1.015	0.080
120	1.027	0.914	0.921	0.076
168	1.026^{a}	0.912^{ab}	0.883^{b}	0.051

Mean values with their SEM (n 6). a, b, c Mean values within a row with unlike letters were significantly different (P < 0·05).

Table S11. Relative protein expressions of occludin in jejunum on three groups at 0, 24, 48, 72, 120, 168 h after placement.

Time after placement (h)	Time of feed deprivation（h）			SEM
	0	24	48	
0	72.69	73.44	74.54	4.709
24	77.81	68.35	79.60	7.369
48	139.3^{a}	87.14^{b}	40.47^{c}	6.948
72	88.80	90.95	92.37	6.081
120	75.71	76.13	65.92	4.448
168	73.78^{a}	58.14^{ab}	43.61^{b}	7.120

Mean values with their SEM (n 6). a, b, c Mean values within a row with unlike letters were significantly different (P < 0·05).

Table S12. Relative protein expressions of claudin-1 in jejunum on three groups at 0, 24, 48, 72, 120, 168 h after placement.

Time after placement (h)	Time of feed deprivation（h）			SEM
	0	24	48	
0	76.59	78.60	80.59	3.440
24	86.87[a]	55.13[b]	62.94[b]	3.039
48	205.4[a]	67.69[b]	40.58[c]	6.660
72	84.12[a]	61.38[b]	53.04[b]	3.845
120	62.26	53.77	64.54	4.500
168	105.4[a]	87.33[a]	46.31[b]	6.320

Mean values with their SEM (n 6). a, b, c Mean values within a row with unlike letters were significantly different ($P < 0·05$).

Table S13. Effects of initial feeding time on growth performance and feed utilization in broilers.

Items	Group			SEM
	A	B	C	
1-7d				
1d BW(g)	42.97	43.03	42.89	0.378
7d BW(g)	123.8[a]	111.6[b]	97.79[c]	1.109
ADG (g/d)	11.61[a]	9.79[b]	7.84[c]	0.161
ADFI (g/d)	14.73[a]	13.95[a]	10.22[b]	0.383
F/G	1.269[b]	1.425[a]	1.303[b]	0.031
1-21d				
21d BW (g)	474.1[a]	455.0[ab]	421.1[b]	14.51
ADG (g/d)	20.53[a]	19.62[ab]	18.02[b]	0.685
ADFI (g/d)	28.45[a]	27.00[ab]	25.76[b]	0.886
F/G	1.386[b]	1.376[b]	1.430[a]	0.015
22-50d				
50dBW(g)	1744	1723	1674	61.51
ADG (g/d)	44.19	43.74	42.84	1.705
ADFI (g/d)	111.6	109.3	106.7	3.124
F/G	2.530	2.500	2.493	0.040
1-50d				
ADG (g/d)	34.02	33.61	32.61	1.229
ADFI (g/d)	74.33	72.90	70.97	2.231
F/G	2.188	2.170	2.178	0.021

Mean values with their SEM (n=6). a, b, c Mean values within a row with unlike letters were significantly different ($P < 0·05$).

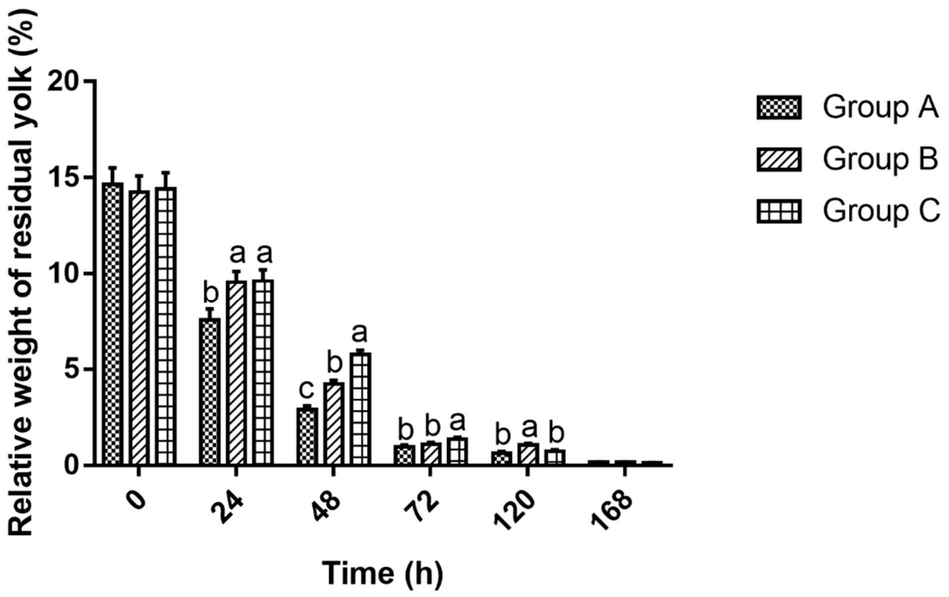

Figure S1. Effects of feed deprivation on relative weight of residual yolk in broiler chicks (n 6). a,b,c Mean values within a row with unlike letters were significantly different (P < 0.05).

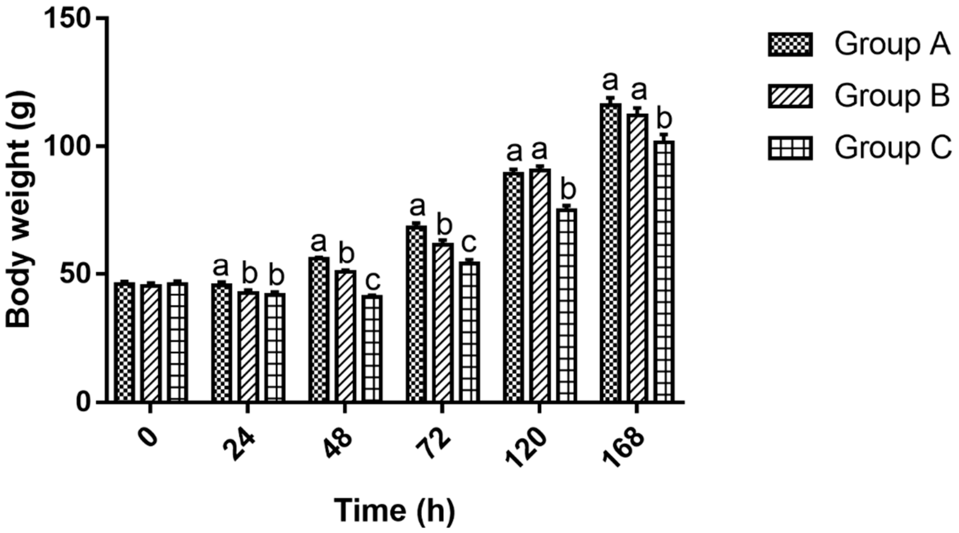

Figure S2. Effects of feed deprivation on body weight. Mean values with their SEM (n=6). a,b,c Mean values within a row with unlike letters were significantly different (P < 0.05).
